# P02.84. A randomized trial of Polarity therapy for stress and pain reduction in American Indian and Alaska Native family dementia caregivers

**DOI:** 10.1186/1472-6882-12-S1-P140

**Published:** 2012-06-12

**Authors:** L Korn, R Logsdon, N Polissar, A Gomez-Beloz, T Waters, R Ryser

**Affiliations:** 1 Capella University and the Center for Traditional Medicine, Puerto Vallarta, Mexico; 2 Dept. of Psychosocial & Community Health, University of Washington, Seattle, USA; 3 The Mountain-Whisper-Light Statistical Consulting, Seattle, USA; 4 Center for World Indigenous Studies, Olympia, USA

## Purpose

Caregivers of individuals with dementia experience elevated stress that places them at increased risk for health problems. The purpose of this study was to compare a complementary/alternative medicine (CAM) method, Polarity therapy (PT), to an enhanced respite control condition (ERC) to reduce stress, depression and pain for American Indian and Alaskan Native (AI) family caregivers. A mixed methods, community participatory, indigenous values approach was combined with a randomized controlled clinical trial to assure both ecological validity and scientific rigor of the investigation.

## Methods

Forty-two AI family caregivers of individuals with dementia, living on and off reservations in the Pacific Northwest, were randomized to an 8-session trial of PT or ERC. PT is a touch therapy that derives from Ayurveda and Cranial Osteopathy to facilitate psychophysiological relaxation and energetic and structural balance. ERC included respite care for the person with dementia and a choice of relaxation (yoga, sauna, basketweaving, etc.) activities for the caregiver. Primary outcome measures included caregiver perceived stress, depression, quality-of-life, sleep quality, worry, and physical health. Average age of caregivers was 50 years (range 27-69); 90% were female; 52% were daughters, 10% wives, 7% sons, and 31% other relatives.

## Results

Baseline 24-hour cortisol demonstrated below normal waking levels in a majority of participants, and 24-hour Heart Rate Variability was significantly lower than the reference population, indicating high levels of chronic stress. Statistically significant reductions in stress, depression, pain and increase in vitality were demonstrated in the PT group, relative to the ERC group. Qualitative data from caregiver narratives provided further insight into phenomenological and spiritual experiences.

## Conclusion

A participatory, multi-methods approach is both feasible and ideal when working with AI caregivers. Caregivers in this sample experienced high levels of chronic stress, and polarity therapy was an acceptable and effective intervention to decrease stress and improve well-being.

